# Super-Treg: Toward a New Era of Adoptive Treg Therapy Enabled by Genetic Modifications

**DOI:** 10.3389/fimmu.2020.611638

**Published:** 2021-02-24

**Authors:** Leila Amini, Jenny Greig, Michael Schmueck-Henneresse, Hans-Dieter Volk, Séverine Bézie, Petra Reinke, Carole Guillonneau, Dimitrios L. Wagner, Ignacio Anegon

**Affiliations:** ^1^ BIH Center for Regenerative Therapies (BCRT) and Berlin Center for Advanced Therapies (BeCAT), Charité-Universitätsmedizin Berlin and Berlin Institute of Health (BIH), Berlin, Germany; ^2^ INSERM, Centre de Recherche en Transplantation et Immunologie, UMR 1064, ITUN, Nantes, France

**Keywords:** immune tolerance, transplantation, autoimmunity, genome editing, CAR, cell therapy, immune regulation, regulatory T cells

## Abstract

Regulatory Tcells (Treg) are essential components of peripheral immune homeostasis. Adoptive Treg cell therapy has shown efficacy in a variety of immune-mediated diseases in preclinical studies and is now moving from phase I/IIa to larger phase II studies aiming to demonstrate efficacy. However, hurdles such as *in vivo* stability and efficacy remain to be addressed. Nevertheless, preclinical models have shown that Treg function and specificity can be increased by pharmacological substances or gene modifications, and even that conventional T cells can be converted to Treg potentially providing new sources of Treg and facilitating Treg cell therapy. The exponential growth in genetic engineering techniques and their application to T cells coupled to a large body of knowledge on Treg open numerous opportunities to generate Treg with “superpowers”. This review summarizes the genetic engineering techniques available and their applications for the next-generation of Super-Treg with increased function, stability, redirected specificity and survival.

## Introduction

The immune system has developed physiological regulatory mechanisms to avoid excessive intensity or duration of immune responses and inflammation. Undesired immune reactivity needs to be controlled in pathological situations such as autoimmune diseases, solid organ transplantation (SOT), graft-*vs*.-host disease (GvHD), and immunogenicity of gene therapeutics and biologics. These regulatory mechanisms can be exploited therapeutically to reshape immune responses in subtler ways than conventional immunosuppressors. In fact, conventional immunosuppressors are non-selective, also inhibit protective anti-pathogen immunity and have common off-target toxicities. Although novel treatments dampen immune responses more specifically and induce immune tolerance (1, 2), alternative treatments are needed.

Among the mechanisms that maintain tolerance, both CD4^+^ and CD8^+^ FOXP3^+^ Treg play a central role (3–7). In addition, in both CD4^+^ and CD8^+^ compartments FOXP3^−^ Treg are described (8). Treg are multifunctional, adaptable, living drugs, that have the potential to restore/induce durable immune tolerance and thus cure or ameliorate diseases as demonstrated in pathological rodent models (3). Although most Treg used in pre-clinical models have been polyclonal, some were antigen-specific or genetically modified (5, 6, 8–10). Small clinical studies have demonstrated the safety and some efficacy of autologous *in vitro* expanded polyclonal CD4^+^ Treg without genetic modifications in a variety of diseases (3).

Genetic modifications hold great potential to enhance their clinical efficacy as previously shown for genetically modified conventional T cells (Tconv) in the cancer field (11). The exponential development of genome engineering approaches enables strategies to generate “Super-Treg.”

This review describes genetic engineering techniques to increase the specificity, functional stability, survival, and suppressive function of Treg, as well as the generation of allogeneic off-the-shelf products, and strategies to eliminate these Super-Treg if necessary.

## Genetic Engineering Tools for the Generation of Super-Treg

Targeted genetic manipulation of Treg has surged due to advances in genetic analysis and engineering (12).

### Gene Transfer Using Lenti-/Retro-Viruses or Transposases

**Figure 1:**
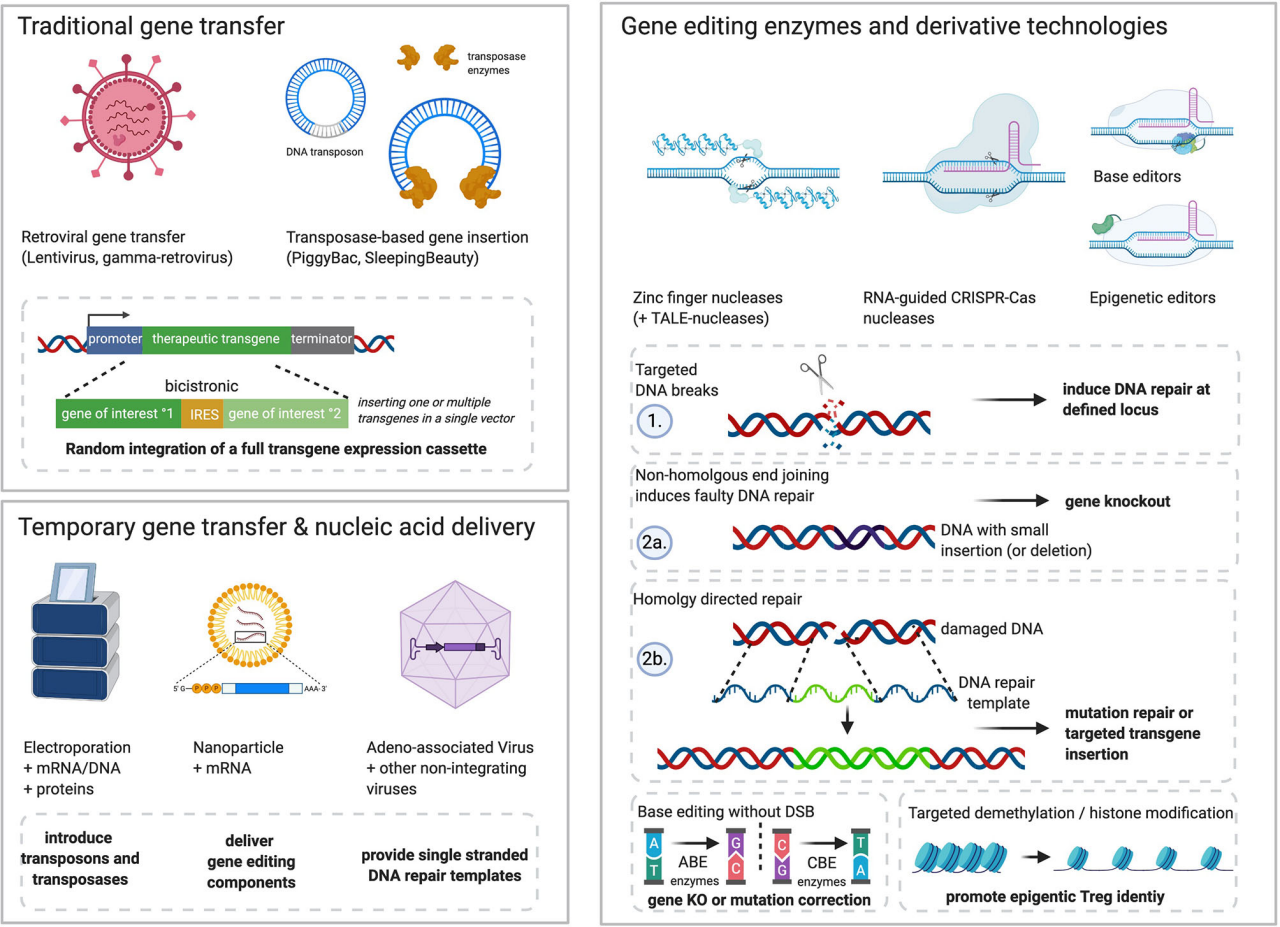
Examples of tools and technologies that allow genetic engineering of Tregs. Traditional gene transfer methods include retroviral transduction or transposase-mediated gene transfer. Random integration of expression cassettes into the Treg genome allow for stable transgene overexpression of one or multiple transgenes connected by internal ribosomal entry sites (IRES) or self-cleaving 2A peptide sequences. Recent advances in the production of plasmids with minimal bacterial backbone (mini-/micro-circles) as well as enhanced transposase enzymes could qualify the use of transposase-modified T cells (52–55), but have not yet been used to generate therapeutic Treg. Gene editing and its derivative technologies allow sequence-specific modification of the human genome. ZFNs and TALE-nucleases bind specific DNA sequences through protein-DNA interaction (zinc-finger arrays, TALE-effectors). Both systems have been used to modify T cell products in preclinical and clinical investigations for HIV or cancer therapy (56–58). CRISPR-Cas ribonucleoprotein complexes can be redirected through small guide RNA (gRNA) and minimal DNA-motif requirements by the Cas enzyme (a.k.a. protospacer adjacent motif, PAM) that are different among Cas variants. After binding of their target sequence, attached or inherent nuclease domains induce DNA double strand breaks (DSB) and subsequently DNA repair. Non-homologous end joining (NHEJ) links the free DNA ends without proofreading, thereby leading to errors like small insertions or deletions that can disrupt genes through frameshifts in their open reading frame. This can be used to knock-out genes and prevent functional protein synthesis. Alternatively, highly activated Treg in S-phase of the cell cycle may also use homology-directed repair (HDR) after DSB. DNA with sequence homology to the cutting site is recognized by the HDR machinery in proximity and used to repair the break *via* proofreading from the analogous DNA fragment. This can be exploited to correct mutations or introduce new genes. To this end, large amounts of single/double stranded DNA templates including desired changes (*e.g.*, nucleotide changes, transgene inserts) must be delivered into the Treg nucleus (typically by electroporation or non-integrating viruses). Important derivative technologies of programmable nucleases include base editors and epigenetic editing enzymes. Base editors are engineered multi-enzymes complexes typically attached to nuclease-deficient Cas proteins which allow targeted modification of certain bases within the gRNA target sequence. Common variants include adenine base editors (=ABE) which convert adenine to guanine (A:T to G:C) and cytosine base editors (=CBE) which convert cytosine to thymidine (C:G to T:A). Furthermore, targeted changes to the epigenome could be performed through enzymes that interfere with methylation or histone modifications to promote desired epigenetic imprints. While retroviral delivery tools benefit from their ancestors’ capacities to invade T cells naturally, other cargo must be effectively delivered into Treg. Electroporation is a common method to transiently introduce nucleic acids like DNA (transposon technology), but also mRNA encoding gene editing enzymes or even recombinant proteins. Nanoparticles are another alternative for transient delivery of gene engineering tools which are under development for Tconv and Treg. Further, adeno-associated viruses (AAV) and other non-integrating viruses may allow a controlled delivery of DNA templates into Treg nuclei for efficient gene targeting. Figure generated using www.biorender.com.

The current gold standard for the stable ectopic gene expression by T cells are replication-deficient lenti-/retro-viruses, which insert entire gene expression cassettes into the genome (13) (**Figure 1**). Multiple studies have demonstrated that Treg from healthy donors and autoimmune patients can be efficiently transduced *in vitro* (**Table 1**) (45). Alternatively, transposon-based gene transfer systems allow the random insertion of moderate to large cargo sizes in T cells (46, 47). Random integration of the genetic cargo and insertional mutagenesis are safety concerns requiring long-term monitoring (48), although, so far no leukemic transformation has been reported for virally transduced Tconv (49–51).

**Table 1:** T cell products modified *in vitro* to treat immune-mediated disease models *in vivo* or cells from human patients with genetic diseases^1^.

Indication		Genetic modification, technology	Results	Reference
**Tissues/SOT**				
Partially mismatched heart transplant in mice		Murine H-2Kd-spec TCR in murine CD4^+^ Treg, γ−retroviral vector	Long-term survival of grafts treated with TCR transduced Treg	(14)
Human skin rejection in NSG mice with human HLA-A2^+^ PBMCs		Human HLA-A2 CAR on human CD8^+^ Treg, lentiviral vector	Prevention of skin rejection, superior to polyclonal Treg	(9)
Human skin xenograft transplant model		HLA-A2-specific CAR on human CD4^+^ Treg, lentiviral vector	Diminished skin pathology, superior to polyclonal Treg	(15)
Human skin (HLA-A2^+^) in NRG mice with human allogeneic HLA-A2^−^ PBMCs		Human HLA-A2 CAR CD4^+^ Treg, γ−retroviral vector	Prevention of skin rejection, superior to polyclonal Treg	(16)
Allogeneic islet rejection in mice		Mouse Treg expressing anti-FITC MAbCAR, lentiviral vector	Incubation of Mab CAR Treg with FITC-labeled mAbs directed against islet antigens prevented islet rejection and generated tolerance	(17)
Human skin xenograft model in mice		Human CD4^+^ Treg, lentiviral vector	CD28 but not 4-1BB co-stimulation increases Treg function	(18)
**GvHD/HSCT**				
Xenogeneic GvHD in NSG mice (also in IPEX patient T cells)		Overexpression of FOXP3 from endogenous loci, HDR of a strong promoter using TALENs and AAV as donors	GvHD suppression	(19)
Xenogeneic GvHD in NSG mice caused by human HLA-A2^+^ T cells		Overexpression in human CD4^+^ and CD8^+^ Tconv of FOXP3 with or without HELIOS, γ−retroviral vectors	GvHD is suppressed, CD4^+^/CD8^+^ T cells expressing FOXP3 and HELIOS more suppressive than each gene alone	(20)
Xenogeneic GvHD in NSG mice caused by human HLA-A2^+^ T cells		10 different 2^nd^ generation HLA-A2 CARs human Treg, lentiviral vector with NGFR	CD28wt A2-CAR provide superior effects; TNFR A2-CARs decrease survival *vs*. negative control	(21)
Xenogeneic GvHD in NSG mice caused by human HLA-A2^+^ T cells		Human HLA-A2 CAR CD8^+^ Treg, lentiviral vector	GvHD is suppressed	(9)
Xenogeneic GvHD in NSG mice caused by human HLA-A2^+^ T cells		Human HLA-A2 CAR CD4^+^ Treg, lentiviral vector	Prevented GvHD	(10)
Allogeneic acute GvHD in mice		Mouse CD4^+^CD25^−^ T cells overexpressing *Foxp3*, lentiviral vector	Prevented GvHD, preserved GVL	(22)
**Inherited genetic diseases**				
HLA-transgenic, FVIII-deficient mouse model (hemophilia A)		FVIII-specific TCR in human CD4^+^ Treg, γ−retroviral vector	Inhibits factor VIII-specific antibody production	(23)
FVIII-deficient mouse model		FVIII-specific human CAR in human CD4^+^ Treg, γ−retroviral vector	Suppressed recall antibody response	(24)
FVIII-deficient mouse model		*Foxp3* expressed by mouse T anti-human FVIII cells, γ−retroviral vector	Inhibits factor VIII-specific antibody production	(25)
IPEX		Human *FOXP3* gene in CD4^+^ Tconv cells, lentiviral vector	Conversion into functional Treg, especially with naïve T cells	(26)
T cells and HSPCs		Introduction of *FOXP3* cDNA by HDR *via* CRISPR/Cas9 RNP and recombinant adeno-associated viral serotype 6 + NGFR tag in human Tconv, HSPCs and Treg as well as patient cells	Endogenous locus gene insertion restores physiological regulation in Tconv; also T cell repopulation in humanized mice with^-^corrected HSPCs	(27)
**Allergy/hypersensitivity**				
OVA-induced and passive anaphylaxis in mice		OVA-fused to antigen receptor signaling domains in murine and human CD4^+^ Treg	Autoantibody producing B are suppressed by BAR Treg, protects from hypothermia if given before OVA challenge	(28)
**Autoimmunity**	**IBD**			
	Tconv cell transfer	Overexpression of *Foxp3*, γ−retroviral vector	Control of intestinal inflammation	(29)
	Trinitrobenzenesulphoric acid colitis in mice	2,4,6-trinitrophenol-specific murine CAR in CD4^+^ Treg, γ−retroviral vector	Reduces acute colitis	(30)
	T cell-transfer colitis and azoxymethane–dextran sodium sulfate model for colitis-associated colorectal cancer	Carcinoembryonic antigen CAR in mouse CD4^+^ Treg, γ−retroviral vectors	Reduces acute colitis in both models and reduction in colon cancer	(31)
	Colitis induced by naïve T cells injected in Rag2−/− mice	TET1-CD overexpression in murine CD4^+^ Treg, γ−retroviral vector	Reduced weight loss	(32)
	**Type 1 diabetes**			
	NOD mice	Foxp3 overexpression in CD4+ Tconv with or without islet specificites, γ−retroviral vector	Only with antigen-specific CD4 cells but not polyclonal CD4+ FOXP3+ controlled recent onset diabetes despite similar suppression *in vitro*	(33)
	NOD mice	Murine CD3ζ/CD28 human insulin-specific CAR and Foxp3 (proteolytic cleavage) + CD90.1 with IRES in naïve CD4+ effector T cells, γ-retroviral vector	Despite effector origin behavior and nTreg like phenotype, unable to prevent diabetes, but survive 17 weeks	(34)
	**Neurological**			
	Experimental autoimmune encephalitis induced by MOG, intranasal Treg delivery	CAR anti-MOG- + murine Foxp3 sequences in CD4^+^ T cells, lentiviral vector	Reduces symptoms, cytokine release; induce resistance to MOG re-challenge	(35)
	Experimental autoimmune encephalitis	MOG or MOG/NF-M bi-specific TCR- murine CD4+ Treg, γ−retroviral vector	Superiority of bi-specific Treg even if disease initiating antigen is not directly targeted	(36)
	Experimental autoimmune encephalitis in DR15 transgenic mice induced by MOG	Myelin-basic protein specific TCR transgenic human CD4+ Treg γ−retroviral vector	Alleviation of symptoms	(37)
	**Rheumatological**			
	Collagen-induced arthritis	TCR from CD4+ cells involved in arthritis expressed in Tconv in association with Foxp3 and TNFR-Ig, γ−retroviral vectors	Inhibition of disease and of inflammatory cytokines	(38)
	Collagen-induced arthritis	Tamoxifen-inducible or constitutive ectopic FOXP3 expression, γ−retroviral vectors	Only inducible FOXP3 expression inhibited the disease due to Treg migration to lymph nodes	(39)
	Arthritis mouse model (immunization with OVA followed by intra-articular rechallenge)	Murine OTII TCR *Foxp3*, γ−retroviral vector	Prevent symptoms when rechallenge combined with OVA	(40)
	Humanized mice (HLA-DR1 transgenic), arthritis induced with bovine collagen II + adjuvant	Foxp3 + HLA-DR1 covalently linked to type II collagen antigen in murine naïve T cells, retroviral vector	Inhibition of disease development and reduction of autoimmune effector T cells	(41)
	Lupus mouse model	Tconv expressing anti-CD19 CARs	B cells are killed, reduction in kidney lupus lesions	(42)
	**Skin**			
	Contact hypersensitivity and autoimmune dermatitis	CD4^+^ Tconv with ectopic expression of Foxp3, γ−retroviral vector	Reduced skin lesions, homing of Treg to skin and lymph nodes	(43)
	Pemphigus mouse model	CD4^+^ Tconv expressing an autoantigen in a chimeric autoantibody receptor (called CAAR) are recognized by autoreactive desmoglein-3 B cells, lentiviral vector	Autoreactive desmoglein-3 B cells are killed by CAAR Tconv, reduction of pemphigus lesions	(44)

**^1^**Only T cells genetically modified in vitro, Treg derived from KO or KI mice are excluded from this table but mentioned in the text.

HSPC, hematopoietic stem progenitor cells; NF-M, neurofilament-medium.

### Gene Editing With Programmable Nuclease Systems

Zinc finger nucleases (ZFNs) and transcription activator-like effector nucleases (TALENs) enable recognition of a genetic sequence through protein/DNA binding and induce double-strand DNA breaks (DSBs) *via* dimerization (74–76) (**Figure 1**). However, the discovery of the CRISPR-Cas system has induced a paradigm shift as it enables easier design of efficient nucleases. Recently, highly efficient optimized Cas9 nuclease variants have been developed (77–79). DSBs at specific sequences are repaired by non-homologous end joining or homology directed repair (HDR) (by providing a DNA repair template) to achieve gene KO or targeted mutation/insertion respectively (**Figure 1**). This was successfully applied to sorted human Treg in the correction of a pathogenic IL-2Ra in approximately 20% and GFP insertions in up to 40% of CD4^+^ Treg (80). Targeting multiple genomic loci with site-specific nucleases allows multiplexing of gene knockouts (KOs) in a single intervention. Two recent manuscripts described CRISPR/Cas9 KO screening in Tregs to define genes involved in mouse Treg stability and function (63, 81).

Nucleases without active nuclease domains can be repurposed to shuttle other bioactive cargo to introduce small base changes, modify epigenetic marks or interfere with transcription (82). Nuclease-deficient Cas9 (dCas9) fused to enzymes with different functions, can be used to specifically edit certain bases (83) (**Figure 1**). Use of base editor proteins for gene multiplexing was successfully achieved with very high efficiency in Tconv (84). Potentially, the newly introduced prime gene editing system could also be applied to insert or replace small gene sequences efficiently without the need for DNA DSBs (85).

### Delivery of Gene Editing Components Into Cells

Gene editing requires efficient delivery of the respective components into the cells’ nuclei. Gene editing enzymes can be transferred as plasmid, mRNA, or recombinant protein-RNA complexes (RNP). HDR repair templates are required as single- or double-stranded DNA. Electroporation allows the highly efficient transfection of protein, mRNA, or plasmids into T cells. Viral vectors exploit their tropism to deliver their cargo with more control than blunt electroshocks. Adenovirus-associated virus (AAV) serotype 6 has been prominently used to deliver genetic cargo into human Tconv and immunopathology-polyendocrinopathy-enteropathy-X-linked (IPEX) syndrome-patient-derived Tconv to induce Treg (27, 86). Lentiviruses and AAVs can be modified to incorporate nuclease enzymes in their capsids to achieve all-in-one delivery solutions for CRISPR-Cas gene editing and DNA transfer tested in mice and human embryonic kidney cells or lymphoblastoid cell lines (87–90). Combination of transposon-based CAR transfer through an anti-CD3 directed nanoparticle system allowed efficient T cell reprogramming in immunocompetent mice *in vivo* (52).

### Potential Genotoxicity of Gene Editing

Off-target effects are a concern for the clinical translation of gene editing and careful experimental design as well as thorough off-target analysis are required (91). Transient presence of the components and high-fidelity nucleases reduce the risk of off-targets. Further, unwanted repair outcomes at the edited on-target sites have been observed including large deletions and translocations (92, 93). Translocations are a particular risk when multiplexing loci in a single manipulation (94), and decrease cellular fitness after transfusion (95).

### Immunogenicity of Cells After Genetic Modification

Viral vectors, nuclease systems, and newly introduced transgenes can be immunogenic, potentially decreasing the efficacy of Super-Treg and even posing a potential safety risk as previously seen using Tconv (96, 97). Of note, most human adults have pre-existing adaptive immunity toward Cas9 proteins and enriched Cas9-reactive Tconv can eliminate Cas9-expressing lymphoblastoid cell lines *in vitro*, which can be reduced by Cas9-specific Treg (98). However, T cells edited using Cas9 delivered by RNP electroporation did not elicit an immune response and persisted, which might be due to the low abundance of Cas9 in the edited final product or defective immune responses in the patients (95).

For TALENs and ZFNs, despite being immunogenic *per se*, stemming from *Xanthomonas*, which infects plants, and partially from *Flavobacterium okeanokoites*, which was isolated from the seabed, there is a low risk for previous exposure to the enzymes. In contrast, Cas9 stems from *Streptococcus pyogenes*, which is a common human pathogen. However, development of an immune response upon permanent expression may still be relevant.

## Genetic Engineering Strategies for Enhanced Stability and Function of Treg

**Figure 2:**
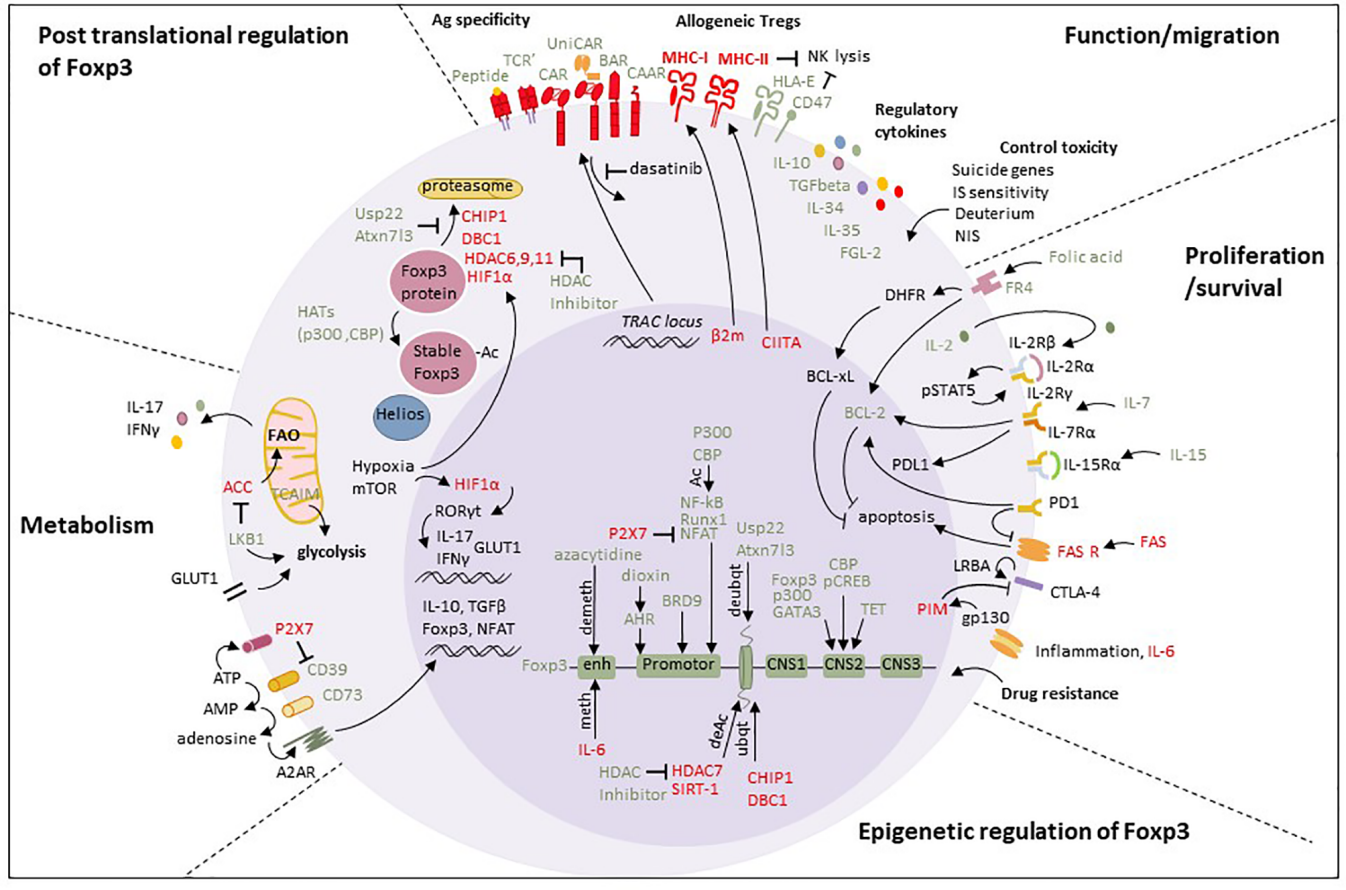
Summary of targets to generate Super-Treg. Foxp3 expression can be modulated at the epigenetic level. Demethylation of CpG dinucleotides at the *FOXP3* locus by azacytidine or TET stabilizes *FOXP3* expression, while remethylation at the upstream enhancer by IL-6 reduces *FOXP3* expression (59). Histone acetylation by HATs (p300, CBP) stabilizes *FOXP3* expression by cooperating with key Treg transcription factors that act on the *FOXP3* promoter, such as Runx1 and NFAT (60). On the contrary, the histone HDAC SIRT-1 inhibits FOXP3 transcription and its deletion promotes Treg function (61). Treg stability is also maintained in inflammatory environments by CBP and P300 interaction with the *Foxp3* CNS2 region, through which CBP is able to regulate pCREB and P300 to regulate expression of GATA3 (60, 62). Histone deubiquitination by Usp22 and Atxn7l3 promotes *FOXP3* expression, contrary to CHIP1 and DBC1. Chromatin remodeling by BRD9, a member of the SWI/SNF chromatin remodeling complex, positively regulates *FOXP3* as BRD9 depletion reduces the binding of *FOXP3* to its enhancers CNS2/CNS0, thereby reducing *FOXP3* expression (63). BRD9 also regulates a subset of *FOXP3* target genes by promoting both *FOXP3* binding at their regulatory element and increasing histone modifications. Knockdown of BRD9 thereby compromises *FOXP3* expression and Treg function *in vitro* and *in vivo* (63). Thus, *BRD9* overexpression could be interesting in Treg. Transcription factor AHR can trigger the differentiation of Treg by the expression of *FOXP3* when activated in response to dioxin, whereas carbazole induces Th17 cell development (64). *FOXP3* expression can also be regulated at the *post* translational level. Acetylation by p300 and CBP stabilizes *FOXP3* protein, while deacetylation by CHIP1, DBC1, or HDAC7 induces degradation of the protein, which can be inhibited by HDAC inhibitors, Usp22 and Atxn7l3. Glycolysis can be privileged. HIF1α binds to the promoter of *RORγt*, resulting in expression of IL-17 which drives Th17 cell differentiation (65) and reducing Treg stability through the production of IFN‐γ (66). HIF1α also increases glycolysis by upregulating GLUT1, and promotes *FOXP3* ubiquitination. LKB1 and TCAIM (67) promoting glycolysis are promising candidates for consideration. CD39 participates in tolerance induction in kidney grafts (68) and the effector memory Treg subset mainly expressing CD39 is diminished in multiple sclerosis (69). CD39 and CD73 transform ATP in adenosine acting through A2AR, which is limited by ATP uptake by P2X7. Function and migration can be controlled. Antigen specificity of Tregs can be modified by inserting genes encoding for an ectopic TCR or a chimeric antigen receptor preferentially in the TRAC locus. The control of rogue Super-Treg could include potent immunosuppressive drug regimens but they require hours to days for elimination. Recently, the small molecule tyrosine inhibitor dasatinib was shown to be a fast and potent inhibitor of CAR signaling in Tconv and may also be applicable to Treg platforms (72). Allogeneic Tregs present advantages regarding production but allogeneic MHC molecules have to be KO and counterbalanced by HLA-E and CD47 (“do not eat me” molecules). The expression of cytokines known to be responsible for Treg function such as IL-10, TGFβ, IL-34, IL-35, and FGL-2 can be upregulated. Treg-mediated toxicity can be controlled by insertion of suicide genes, such as a truncated version of the epidermal growth factor receptor recognized by the mAb cetuximab (70), or the peptide RQR8 combining epitopes recognized by the mAb rituximab (71) or by dimerization of Casp9 by the small molecule rimiducid (72). Tregs can be tracked using deuterium and NIS. Proliferation and survival can be promoted. Folic acid is suggested to upregulate the anti-apoptotic proteins BCL-2 and BCL-xL *via* folic acid receptor 4 (FR4) in Treg (73). Hence, increased expression of FR4 or enzymes of this pathway such as dihydrofolate reductase in Treg may preferentially preserve these cells. IL-2, IL-7, and IL-15 signals are important for survival and proliferation of Tregs. To make Treg independent of exogenous IL-2, they could be armed with their own IL-2 for self-supply. PD1 and CTLA-4 have both important roles for Treg function and survival and LRBA, in contrast to PIM, is important for CTLA-4 expression. Drug resistance could also be considered for promoting Treg survival.

A limitation of adoptive Treg therapy is that inflammatory conditions can inhibit their function or even switch them to Tconv (99, 100). Therefore, the identification of pathways regulating Treg function and stability are key to define targets for genetically engineering more stable and robust Treg (**Figure 2** **)**.

### FOXP3

Given the key role of FOXP3 to control Treg function and that its expression and function are labile or even lost in patients with mutations in the *FOXP3* gene (IPEX), numerous studies have analyzed how to increase or stabilize its expression. Functional CD4^+^ Treg from IPEX patients could be obtained by ectopic expression of FOXP3 in their Tconv (26), but also with more clinical potential by precise HDR on hematopoietic stem cells (27) (**Table 1**).

This strategy could also allow high numbers of Treg to be obtained from Tconv. In CD4^+^ Tconv, ectopic expression of FOXP3 using retroviral vectors (22, 26, 43) or by HDR of a strong promoter upstream of the FOXP3 coding sequences (19) allowed generation of Treg that suppressed CD4^+^ Tconv not only *in vitro* but also inhibited GvHD, colitis or dermatitis in animal models (**Table 1** **)**. Interestingly, tamoxifen-induced but not constitutive FOXP3 expression in CD4^+^ Tconv resulted in control of autoimmune arthritis by migration into lymph nodes (39). In line with these results, control of type 1 diabetes (T1D) was obtained only with islet-specific CD4^+^ Tconv homing to lymph nodes and not with polyclonal CD4^+^ FOXP3-expressing Tconv in a mouse model (33). Transduction of mouse anti-human FVIII T cells with *Foxp3* resulted in decrease anti-FVIII antibodies in hemophilia A mice (25). In *in vitro* studies, human pathogenic synovial Tconv from rheumatoid arthritis patients ectopically expressing FOXP3 showed reduced Tconv responses (101). Other publications with *in vitro* studies described that ectopic expression of one (102) or both (103) isoforms of FOXP3 in CD4+ Tconv resulted in functional human Treg. Overexpression of the transcription factor HELIOS cooperates with FOXP3 to generate both CD4^+^ and CD8^+^ Treg from human Tconv, but particularly CD8^+^ T cells (20). Similarly, delivery of dCas9 fused to a transcriptional activator and guides recognizing *FOXP3* promoter sequences increases FOXP3 expression (104).

Epigenetic regulation of *FOXP3* expression is important for the expression of FOXP3 in Treg. Demethylation of CpG dinucleotides at the *FOXP3* locus including at regulatory elements in the intronic region, at the proximal promotor and the upstream enhancer stabilizes *FOXP3* expression (59, 105, 106). If these Treg-specific demethylated regions (TSDR) are not fully demethylated, such as in the induction of *FOXP3* expression by TGF-β, FOXP3 expression can be lost upon restimulation in mouse Treg (107). Epigenetic modifications of *FOXP3* for TSDR demethylation in Treg by azacytidine (a DNA methyltransferase inhibitor) induces and stabilizes *FOXP3* expression in mouse Treg (107). Partial demethylation of TSDR CNS2 in Treg by catalytically inactive CRISPR-Cas9 (dCas9) fused to the catalytic domain of ten-eleven translocation (TET) protein, which promotes demethylation, resulted in stable *FOXP3* expression and increased suppressive activity *in vivo* in mice (32). However, a similar system using dCas9 fused to TET1 did not increase FOXP3 levels in mouse Treg (108).

Transcription of *FOXP3* can be repressed by histone deacetylation in the *FOXP3* promoter by histone deacetylase 7 (HDAC7), and HDAC inhibitors increase *FOXP3* expression through regulation of both the gene and the protein, and can improve the suppressive action of murine and human Treg (109, 110). A dCas9 fused to the catalytic domain of histone acetyltransferase (HAT) p300 showed that histone acetylation targeted to the promoter locus was able to activate and stabilize FOXP3 levels in mice, even under inflammatory conditions (108).

Lysine acetylation by HATs of both histones in the *FOXP3* locus and of FOXP3 itself increases its transcription and reduces its poly-ubiquitination and degradation as well as enhancing FOXP3 chromatin binding in mouse and human Treg (111, 112). Hyperacetylation by HDAC inhibitors or overexpression of HATs can increase FOXP3 levels in mouse and human Treg (112, 113). For example, P300 and CBP HATs acetylate FOXP3, increasing its DNA binding and thereby regulating murine Treg function and stability (60). CBP and P300 affect Treg development through several mechanisms, including promoting FOXP3 production, and by participating in a positive feedback loop that enhances murine Treg stability in inflammatory environments, which could be further exploited through molecular engineering (60).

Ubiquitination of both histones at the *FOXP3* locus and of the protein itself is important in the regulation of FOXP3, *via* members of the deubiquitination module of the SAGA complex, Usp22, and Atxn7l3. Loss of Usp22 in Treg reduces *Foxp3* transcript levels, increases FOXP3 ubiquitination and degradation, and reduces suppressive activity *in vivo* in mice (63, 81). Furthermore, Stub1 (114) and TRAF6 (115) E3 ubiquitin ligases induced by inflammation target the ubiquitination of FOXP3 followed by its degradation in mouse and human Treg and represent interesting targets for genetic ablation in Treg products. In contrast, Hrd1, an E3 ligase critical in suppressing the ER stress response, stabilizes murine FOXP3 expression (116). In terms of kinases and phosphorylation of FOXP3, PIM1 (117), and CDK2 (118) kinases negatively regulate FOXP3 and Treg function.

### Other Transcription Factors

HIF‐1α reacts to hypoxia by triggering the switch between mitochondrial oxidative phosphorylation and aerobic glycolysis (119), and is also induced by continuous TCR stimulation *via* mTOR in human T cells (120). In mouse Treg and human embryonic kidney cells, HIF-1α promotes the ubiquitination and proteasomal degradation of FOXP3 (65), and its upregulation in response to hypoxia inhibits FOXP3 expression in mouse T cells (121). HIF-1α also inhibits the development of mouse Treg through increasing glycolysis by upregulating glycolytic proteins (122). HIF-1α deficiency inhibits glycolysis and therefore promotes the differentiation of murine Treg over Th17 cells (123). Deletion of HIF-1α in mice increases FOXP3 expression, and reduces transcription of Th17 cell-related genes (65, 121), suggesting HIF-1α KO as a means to improve Treg stability through metabolic control. Differentiation of induced human Treg is inhibited by IL-1β in a HIF-1α-dependent manner (124). However, in human Jurkat cells, HIF-1α induction increased FOXP3 protein and mRNA levels, which was reversed by knockdown of HIF1α (125, 126). On the same lines, exposure of human PBMCs to hypoxia increased the proportions of FOXP3^+^ Treg among CD4^+^ CD25^+^ T cells and their suppressive potential to inhibit Tconv proliferation, which was also observed in mouse splenocytes (125).

Aryl hydrocarbon receptor (AHR) is a ligand-dependent transcription factor that functions as an environmental sensor and mediates the differentiation of both Th17 cells and FOXP3^+^ Treg. AHR is highly expressed in peripheral Treg in the gut, and its deletion impairs their function. Conversely, activation of AHR in transgenic mice increases the population and migration of Treg (127). AHR inhibited proinflammatory cytokines (IFNγ and IL-17) and Th1-associated genes, but was dispensable for *FOXP3* stability. *Ahr* activation in a conditional knock-in in Treg in a mouse model of colitis enhances suppressive activity and migration to the inflammatory site, and a reduction in proinflammatory T cells (127). Furthermore, AHR activation was found to promote generation of human induced Treg, producing IL-10 and controlling Tconv *via* granzyme B, but did not have an effect on thymic-derived human Treg (128, 129).

### Pro-Inflammatory Cytokines and Extracellular Metabolites

IL-6 is a proinflammatory cytokine that induces the expression of kinase PIM1 during inflammation, which inhibits expression of Treg markers including CTLA4 and CD25 *via* phosphorylation of FOXP3 (130). T cell-specific deletion of the IL-6 receptor gp130 in mice reduces IL-6 signaling and promotes the conversion of peripheral Tconv into Treg (131). IL-6 blockade suppresses the immune response in models of autoimmune disease and is used in the treatment of rheumatoid arthritis (132, 133). At the same time, a recent publication showed that IL-6Rα-deficient Treg lost suppression and aggravated experimental glomerulonephritis (134). Therefore, mitigating IL-6 signaling in Treg, which was assumed to be a compelling strategy to enhance their functionality in inflamed tissues and in the presence of high levels of IL-6 needs more investigation and has to be overthought critically.

The purinergic receptor P2X7 induces T cell activation through binding of ATP, pushing the balance toward proinflammatory Th17 cells, and decreasing the viability and suppressive function of mouse Treg (135). In a mouse model of experimental colitis, P2X7 receptor KO resulted in an increase of activated Treg, IL-10, and TGF-β (136). Preventing P2X7 signaling is able to preserve mouse Treg stability by stabilization of nuclear complexes of NFAT and FOXP3, and the resulting downstream transcription of Treg-linked genes (135). CD39 and CD73 expressed by Treg degrade ATP to adenosine and adenosine itself can enhance the expansion and immunosuppressive function of human Treg *in vitro* by binding to the purinergic P1 adenosine 2A receptor (A2AR) (137, 138). Thus, genetic overexpression of CD39 could be beneficial.

Although Treg produce immunosuppressive cytokines, such as IL-10 (139), TGFβ (140), IL-34 (141, 142), IL-35 (143), and FGL2 (144, 145), their production could be increased by genetic means.

Treg with increased function and stability could therefore be engineered by inhibition of negative regulatory genes (using nucleases), overexpression of positive regulators (using lentiviral vectors), likely giving more precise control than small molecule treatments which may bind multiple members of a family (for example acetylases).

## Genetic Engineering Strategies for Increased Proliferation and Engraftment of Treg

### Proliferation Signals

Survival of human naïve CD4^+^ Treg is mediated by IL-7 signaling, which increases anti-apoptotic BCL-2 (146) while long-term survival of CD4^+^ Treg is dependent on IL-2 (147) and of CD8^+^ Treg on IL-15 (4). Low dose IL-2 infusion was shown to increase Treg numbers, FOXP3 expression and lead to a more diverse repertoire of CD4^+^ and CD8^+^ Treg in patients (4, 148). Currently, there are different engineered IL-2–based drugs targeting CD25 on Treg, also referred to as IL-2 muteins (149) in clinical trials. Since IL-2 signaling is associated with long-term survival of human Treg, constitutively active STAT5 (150), which is an important signal transducer in this pathway, may improve Treg survival and abolish their dependence on extracellular IL-2 (**Figure 2**). Moreover, to make Treg independent of exogenous IL-2 that may activate Tconv and NK-cells, they could be armed with their own IL-2 for self-supply. However, ectopic IL-2 expression could compromise the immunosuppressive mechanism of IL-2 deprivation of surrounding Tconv. A mutated IL-2R that only binds an IL-2 mutein and not wild-type IL-2 could be engineered in mouse Treg that are then selectively expanded (151).

A strategy to engineer constitutively active cytokine receptors independent of cytokine availability may also be translated from Tconv to Treg (152), *e.g.*, allowing long-term survival *via* a constitutively active IL-2 receptor (147). Moreover, chimeric cytokine receptors (CARs) converting pro-inflammatory signals (captured by the extracellular domain of the respective receptor, *e.g.*, IL-6) into Treg-survival signals using the intracellular signal transduction domains (*e.g.*, IL-2 receptor) of pro-survival signals may contribute to improved survival of Treg products as reported previously in a mirroring approach for the support of Tconv (153).

### Apoptotic Mechanisms

Several pathways inducing Treg apoptosis seem to be dependent on FAS (154, 155) and pro-survival pathways on *BCL-2* (73, 146, 154). Thus, disruption of *FAS* or over-expression of *BCL-2* may significantly increase the viability of Treg. An alternative may be to increase the PD1-PDL1 signaling in Treg, since PD1 blockade was reported to lead to downregulation of BCL-2 and increased FAS receptor expression (154). However, mouse Treg lacking PD-1 were shown to be activated and have high suppressive potential (156), which underlines the necessity for further studies of this axis in human Treg. Additionally, human CD4^+^ Treg express PD-L1 in response to IL-7 (154) and induce apoptosis in PD-1^+^ Tconv (157) and autoreactive B-cells (158). As CTLA-4 has an important role in Treg function and increased degradation of CTLA-4 as present in LRBA deficiency is associated with high levels of Treg apoptosis, stabilizing strategies for sustained or increased CTLA-4 expression may also improve human Treg survival (159, 160).

### Metabolism

Treg and Tconv have different metabolic requirements, as Treg use glycolysis and increase fatty acid oxidation upon activation (161, 162). Acetyl CoA carboxylase (ACC) regulates both biosynthesis and breakdown of long chain fatty acids and ACC1 deficiency induces high levels of FOXP3 expression in mouse and human Treg (163). Therefore, ACC is a potential target for altering the metabolic programming of T cells, as blocking fatty acid synthesis favors Treg induction and prevents Th17 development. Liver kinase 1 (LKB1), a metabolic sensor, is essential for murine Treg stability and suppressive activity by inhibiting expression of pro-inflammatory cytokines and preventing exhaustion (164). *LKB1* and its target genes are downregulated in impaired Treg from patients with acute GvHD and *Lkb1* deletion in Treg in mice leads to severe autoimmune inflammation, and can aggravate acute GvHD (164, 165). LKB1 also stabilizes *FOXP3* expression in Treg and expression levels correlate with Foxp3 expression in human Treg (165). In contrast to Tconv, murine and human Treg do not accumulate lactate and are insensitive to lactate in medium (166). LKB1 increases glycolysis and lactate formation and in mice, abrogation of *Lkb1* leads to loss of mitochondrial integrity and to a dramatic reduction of Treg (167). Thus, LKB1 overexpression could be used to stabilize FOXP3 expression, maintain metabolic homeostasis, and avoid exhaustion in Treg.

### Drug Resistances

In several settings, Treg are infused into patients treated with immunosuppressants to inhibit Tconv but also compromise Treg function (168). Hence, making Treg resistant to these drugs may allow their preferential survival. Indeed, strategies aiming to make antiviral T cells resistant to calcineurin inhibitors or glucocorticoids including knockdown (169), knockout (170–172) or introduction of calcineurin-resistant mutants (173), might also be applied to Treg.

## Genetic Engineering Strategies for Redirecting Treg Antigen Specificity

Treg-mediated tolerance can be improved by increasing antigen specificity and with the development of gene editing, redirection of Treg specificity became feasible (**Figure 2**). Indeed, antigen-specific Treg have an increased suppressive ability and a stronger efficacy in the regulation of the immune response and an improved migration to the site of interest compared to polyclonal Treg (9, 10, 174–178). While *ex vivo* expansion in presence of the antigen of interest, or *in vivo* by administration of peptides recognized only by Treg (176, 177, 179) is possible, using genome editing would be advantageous as it would confer antigen-specificity to a larger Treg population rather than amplifying a very small subset of antigen-specific Treg, which can be challenging. However, genome editing to redirect specificity would also multiply the danger of contaminating Tconv that may have a proliferative advantage in cytokine-rich medium used during Treg expansion and could overgrow the culture, multiplying their abundance in the final product. Thus, a very pure starting population is required or the undesired cells (such as CD8^+^ non-Treg) must be depleted at a later time point. However, the latter is challenging for, *e.g.*, CD4+ Tconv, which are not easily distinguishable from Treg after culture, but could also cause detrimental effects. Furthermore, it will be crucial to choose a receptor with appropriate affinity for Treg to exclude the possibility of instabilities. Even pre-selection of more stable Treg subsets or genetic engineering to make them more stable may be required to generate a safe product.

Treg specificity can be redirected by the use of TCRs and CARs (180, 181). Importantly, Treg with a single specificity have been shown to suppress multiple antigens if presented by an APC simultaneously, as shown in autoimmune, GVHD, and SOT models (9, 10, 15, 182).

### TCRs

Redirecting T cell specificity with an engineered TCR was reported as early as 1996 using a chimeric TCRβ chain consisting of a single-chain Fv portion derived from a monoclonal antibody paired with endogenous TCR/CD3 component, thus providing antibody and TCR specificity (183). The use of TCRs has several advantages since it represents a physiological way of activating T cells and allows the targeting of intracellular antigens presented by HLA molecules. In addition, expression of only one antigen per cell is sufficient to activate the TCR-expressing Treg. However, HLA restriction limits coverage to a particular part of the population. Careful identification of a high affinity TCR-α/β is required to ensure that they retain functionality without acquiring a harmful unpredicted specificity when mispaired with the endogenous TCR. To avoid this, disruption of the endogenous TCR using nucleases might be necessary (184). Proofs of concept include human Treg expressing a myelin basic protein-specific TCR derived from a multiple sclerosis patient, which showed *in vitro* and *in vivo* efficacy in an EAE model (37). Efficacy was also demonstrated in a mouse model of hemophilia A using human Treg engineered with a factor-VIII-specific TCR isolated from an hemophilia A patient (23). TCRs against autoantigens have also shown *in vivo* efficacy in models of arthritis (38) (**Table 1** **)** or *in vitro* recognizing islet antigens involved in T1D (182, 185).

Interestingly, MHC-I-restricted TCRs have been shown to be functional in human CD4^+^ Treg, bypassing the need for the CD8 coreceptor, and this was the case for TCRs with low affinity not functional on CD4^+^ Tconv (186).

A potential future perspective of these studies is the use of TCRs isolated from Treg and not from effector T cells as done until now. Although only very few TCRs and the peptides recognized by Treg TCRs have been identified until now, they do show differences with TCRs from Tconv, *e.g.*, recognizing longer (15aa) peptides or reversed TCR docking modes (176, 177, 187).

### CARs

Pioneer work by Eshar and colleagues in the autoimmune field allowed the generation of CARs in which antigen recognition signaling domains of antibodies and a TCR-zeta-chain were fused in a single molecule (181). Sequences from co-stimulatory proteins were also fused in cis and the most commonly used ones are the intracellular portions of CD28 or 4-1BB. While the 4-1BB signaling domain used in the CAR construct has been suggested to enhance Tconv persistence and improve the toxicity profile in patients, CD28 was shown to be more beneficial for Treg phenotype and function (18). CAR technology has some advantages over TCRs, the most important one being the absence of HLA restriction. CAR-Treg are less dependent on IL-2 compared to TCR-expressing Treg, potentially due to costimulatory signals received upon activation of the CAR. However, CARs also have several limitations *vs*. TCRs, as CARs only recognize extracellular antigens. Additionally, CARs require higher expression levels than TCRs (100–10,000 antigens/cell *vs*. <10, respectively) for sufficient activation although increasing the affinity of CARs can also increase efficacy (188, 189). CAR molecules can be immunogenic, not only due to murine scFv fragments, but also due to the generation of new epitopes in a chimeric molecule, and this impacts the persistence of effector T cells in patients (97, 190). Even induction of anti-CAR antibodies was described for effector T cells and the immune reaction was reported to be able to cause anaphylaxis in a patient repetitively treated with CAR T cells (191). Possibly, anti-CAR immune responses may be less severe if the CAR is expressed in an immunosuppressive Treg compared to expression in pro-inflammatory Tconv.

Human CD4^+^ and CD8^+^ CAR-Treg have been used in mouse models of FVIII hemophilia, SOT and GVHD as well as *in vitro* with CD4^+^ Treg from IPEX patients (for a complete list see **Table 1** **)**. Mouse CD4^+^ CAR-Treg have demonstrated efficacy in mouse models of SOT, GvHD, IPEX, colitis, allergic asthma, rheumatological diseases, and EAE (**Table 1**).

The importance of internal *vs*. external antigen targets could orientate toward the generation of a TCR- *vs*. a CAR-transgenic Treg. There are also new tools developed such as CAR-T cells possessing a TCR-like antibody moiety (TCR-like CAR-T) with a single-chain variable domain specific for a distinct peptide/MHC (192). In an original approach, CD4^+^ Treg expressing a CAR directed against FITC and *ex vivo* incubated with FITC-labeled antibodies directed against donor alloantigens inhibited pancreatic islet rejection (17). Similarly, the UniCAR system, in which a universal CAR is indirectly linked to their target cells *via* a separate targeting module, has been applied to human Treg (193). In a new approach to treat autoantibody-driven diseases, CD4^+^ Treg have been engineered to express CARs with antigens recognized by B-cells (called BARs, where the scFv fragment is replaced by an antigen) (28). Similarly, Tconv expressing an autoantigen in a chimeric autoantibody receptor (CAAR) mediated killing of the autoreactive B-cells, as shown in pemphigus (44). Also, Tconv expressing anti-CD19 CARs generally used to treat B-cell malignancies were used to treat mice with lupus disease (42) (**Table 1**).

## Genetic Engineering Strategies for the Use of Off-the-Shelf Allogeneic Treg

Genetic engineering of allogeneic Treg as an off-the-shelf product would allow cells from a given donor to treat several patients thereby reducing the cost per dose as well as increasing treatment flexibility (**Figure 2**). Nevertheless, this approach has the draw-back of allogenicity due to recognition of foreign MHC-I and -II antigens by host T cells. To extend allo-Treg persistence, deletion of β-2 microglobulin and CIITA could be performed to eliminate MHC-I and -II antigens, respectively. Although absence of MHC-I may increase the susceptibility of Treg to NK cell lysis and that could limit therapeutic efficacy, activated Treg may be more resistant *in vivo* and indeed triple-KO T cells have been found to persist better than HLA-sufficient T cells. In addition, overexpression of HLA-E or CD47, important in NK cell inhibition through inhibitory receptors, could prevent NK cell-mediated lysis (194–196). Preventing an immune response against allogeneic cells is even more important in the Treg setting than in the Tconv setting, as here any pro-inflammatory immune response can be detrimental as opposed to the Tconv setting, in which the goal is to create a pro-inflammatory environment. However, it has also to be considered that Treg have anti-inflammatory properties *per se* and first applications of 3^rd^ party-derived Treg after umbilical cord stem cell transplantations did not reveal relevant adverse events (197).

Another potential risk using allogeneic T cells is GvHD although the inherent suppressive function of Treg makes this risk less relevant than with allogeneic Tconv. Generating highly specific, allogeneic Treg products also harbors the risk of toxicity in the case of unstable Treg or contaminating Tconv. Strategies such as suicide genes and elimination markers—some already clinically evaluated in Tconv—could be included to shut off adoptively transferred “stealth” Treg in case of toxicity (198) (**Figure 2**).

Pluripotent stem cells-derived Tconv mainly for cancer use have been described (199, 200) and derivation of Treg would be an important step not only to have an unlimited source of cells but also for generating “stealth” Treg.

It will also be important to better understand the migration of Treg to different anatomical compartments and their survival. To date, Treg infused in patients have only been identified *in vivo* in T1D patients after labeling CD4^+^ Treg with deuterium but this strategy is limited to cells in circulation and not in tissues (201). Also, mouse CD4^+^ Treg have been transduced for the expression of the sodium-iodide symporter (NIS). NIS uptakes into only living cells plasma iodide and other substrates detected using PET or SPECT/CT. NIS-expressing Treg radiolabeled with Technetium-99m pertechnetate were detected in spleen with no effects on cell viability, phenotype, and function (202).

## Gmp Compliant Manufacturing and Clinical Perspectives of Super-Treg

An increasing number of clinical trials employing adoptive Treg transfer are currently ongoing or registered addressing a large variety of applications (3, 5, 6). The generation of Super-Treg with genetic modifications will require the use of improved protocols for the purification and amplification of Treg to prevent contaminating Tconv with putative hazard. Bead-based or flow cytometry-based cell sorters (9, 14–16, 18), either fully closed or open systems, allow for clinical grade CD4^+^ Treg isolation. For clinical-grade CD8^+^ Treg isolation flow cytometry-based approaches are used. Typically, *in vitro* Treg expansion before adoptive transfer takes around 2–3 weeks (9, 14).

Release criteria usually include the classical phenotypic Super-Treg markers (*e.g.*, CD25, FOXP3 for CD4^+^ Treg) and absence of pro-inflammatory markers, e.g., pro-inflammatory cytokine production and CD45RC for CD8^+^ Treg. Functional assays or epigenetic assays may be beneficial, however cannot be realized in a timely manner before Treg product infusion.

Super-Treg quality control will require additional control of nuclease delivery and duration of expression as well as maximization of efficacy preferentially using vector-free systems (41). Safety controls will include *in silico* as well as *in vitro* analysis of off-target effects of nucleases and careful analysis of the edited loci (41). In this context, a clinical trial using CRISPR/Cas9-genetically modified in cancer patients has been recently published (95).

A Phase I/II clinical trial is approved in UK that plans to apply CAR anti-HLA-A2 CD4^+^ Treg in kidney transplantation. Academic multi-center consortia (like the ReSHAPE consortium, http://www.reshape-h2020.eu/partnership) aim to generate CD4^+^ and CD8^+^ Super-Treg and to apply them to both animal models of immune-mediated disease and clinically in kidney transplanted patients.

## Discussion

The specific challenge of using Treg therapy in general in human pathologies will be to interfere with established autoimmunity, rather than *de novo* immunizations (SOT, GvHD, gene therapies, biologics), without provoking global immunosuppression.

A future direction is the use of CARs recognizing inflamed or damaged tissues that could direct the Treg to these pathological areas, as shown by preliminary data (203).

The demonstration that Treg can stimulate tissue regeneration (204–208) reveals regenerative medicine as a novel indication for Super-Treg.

As for CAR-Tconv (209), the simultaneous use of both CD4^+^ and CD8^+^ CAR-Treg may prove to be superior to each subset alone.

Treg will likely be modified using new T cell engineering strategies, such as synthetic Notch receptors that have an extracellular single-chain antibody and intracellular transcriptional domains that are released and activate expression of target genes (210).

Immune humanized immunodeficient animal models will continue to be useful to address many questions in preclinical studies (211). Moreover, human and/or patient organoids, may gain more importance and are promising candidates for examining Treg function in disease models (212).

Biomarker studies will be important to define not only the effects of Super-Treg therapy but also the timing and doses of their administration.

The knowledge of Treg biology, their success in animal models and early clinical trials as well as the explosion of genome editing techniques are synergistic approaches to treat immune-mediated diseases in the future.

## Author Contributions

DW, JG, LA, SB, CG, and IA performed bibliography searches and wrote and edited the manuscript. MS-H, H-DV, and PR edited the manuscript. DW generated **Figure 1**. SB generated **Figure 2**. LA generated **Table 1**. IA planned the manuscript. All authors contributed to the article and approved the submitted version.

## Funding

This work was funded by the ReSHAPE project from the European Union's Horizion 2020 research and innovation programme under grant agreement No 825392. It was also realized in the context and partially funded by the Labex IGO program supported by the National Research Agency *via* the investment of the future program ANR-11-LABX-0016-01, and a Berlin Institute of Health (BIH) crossfield grant “regulatory T cells.”

## Conflict of Interest

CG, IA, and SB have patents that have been licensed to TxCell S.A., a Sangamo Company. DW, LA, PR, H-DV, and MS-H have filed patent applications on Treg-related diagnostics and therapeutics. The remaining author declares that the research was conducted in the absence of any commercial or financial relationships that could be construed as a potential conflict of interest.
